# Impedance Spectroscopy of Hierarchical Porous Nanomaterials Based on por-Si, por-Si Incorporated by Ni and Metal Oxides for Gas Sensors

**DOI:** 10.3390/s22041530

**Published:** 2022-02-16

**Authors:** Anton Bobkov, Victor Luchinin, Vyacheslav Moshnikov, Svetlana Nalimova, Yulia Spivak

**Affiliations:** Department of Micro- and Nanoelectronics, Saint Petersburg Electrotechnical University “LETI”, Professor Popov Str. 5, 197376 St. Petersburg, Russia; cmid_leti@mail.ru (V.L.); vamoshnikov@mail.ru (V.M.); sskarpova@list.ru (S.N.); ymkanageeva@yandex.ru (Y.S.)

**Keywords:** impedance spectroscopy, gas sensor, fractal, percolation, zinc oxide, porous silicon, adsorption gas sensors

## Abstract

Approaches are being developed to create composite materials with a fractal-percolation structure based on intercalated porous matrices to increase the sensitivity of adsorption gas sensors. Porous silicon, nickel-containing porous silicon, and zinc oxide have been synthesized as materials for such structures. Using the impedance spectroscopy method, it has been shown that the obtained materials demonstrate high sensitivity to organic solvent vapors and can be used in gas sensors. A model is proposed that explains the high sensitivity and inductive nature of the impedance at low frequencies, considering the structural features and fractal-percolation properties of the obtained oxide materials.

## 1. Introduction

Nowadays, monitoring of the air environment is an important task as transport and industrial facilities increasingly pollute it. There are various gas sensors such as thermocatalytic, optical, resistive, electrochemical, etc. The most sensitive ones are optical sensors [1], however, their wider use is limited by high cost. In terms of availability, the most attractive are semiconductor gas sensors. That’s why conductometric gas sensors are among the most widely studied and promising devices for detecting gases such as H_2_S [2,3], H_2_ [4,5], volatile organic compounds [6,7,8], NO_2_ [9,10] etc. Their working principle is based on the measurements of changes in the conductivity of the sensor layer when exposed to the detected gas. For typical n-type semiconductor, it is caused by the interaction with adsorbed gas molecules and the change in the concentration of electrons in the conduction band [11,12]. First, in ambient air, oxygen particles adsorb on the surface of the sensor layer. If the electron affinity of the adsorbed oxygen atom/molecule is higher than the work function of the semiconductor, then the electrons will transfer from the semiconductor to the adsorbed oxygen particles. Thus, the bands will bend in the sub-surface area. The thickness of depleted region depends on the Debye length. The mechanism of the reversible detection process is the chemical reaction between the gas molecules and adsorbed oxygen ions. As a result, the electrons transfer into the conduction band and the reaction products leave the surface in a neutral form. Thus, the concentration of target gas defines the difference in the conductivities of the sensor layer in the air and in the presence of gas molecules [13,14,15].

Conductometric sensors are simple, compact, cost-effective and easy-to-manufacture [16]. At the same time, an increase in their response is required for detecting low concentrations of target gases. Design of new material structure is a promising way to solve this problem. The most interesting approach is the development of materials with a fractal structure [17]. Fractal TiO_2_ gas sensor has excellent performance in acetone detection, which attributed to the high film porosity and fractal like network structures [18]. The processes of fractal structure formation have been considered in SiO_2_/SnO_x_/CuO_y_ nanofilms prepared by the sol–gel method [19]. Maximum NO_2_ sensitivity appears at the ratio Sn/Cu = 6, when the processes of self-organization seem to be more active, resulting in transition of one type of fractal structure to another. ZnO/NiO fractal-percolation nanosystems were developed in [20]. From the example of methane and methanol, it has been shown that based on the character of the I–V curves it is possible to distinguish between different reducing gases. The opening or closing of the current flow channels occur due to the balance between physical and chemical processes at the nanosystems active centers in the presence of reducing gases and oxygen. An increase in sensor response of materials with fractal-percolation structure is achieved when the system in the form of a percolation cluster passes through the percolation threshold when interacting with gas [21].

Porous silicon (por-Si) is very promising as a matrix material for gas sensors due to its high surface to volume ratio and reactivity with the environment as well as its hierarchical porous texture with different pore sizes that can be tuned for certain goals and also due to facility of integration with silicon microchips [22,23,24,25,26].

It was shown that por-Si has a potential use in sensing of organic vapors and ammonia when covered with an oxide layer [22]. Graphene decorated por-Si showed enhanced sensing property to low-ppm H_2_ at room temperature in comparison to that of pristine por-Si [23]. The por-Si substrate with a large specific area was a sensor matrix and graphene was a catalyst material. Compositions based on porous silicon matrices intercalated with nickel and/or nickel oxide are also promising for “multi-response” sensor structures [27,28,29,30,31]. In this case, the properties (catalytic, magnetic, electrophysical, optical, etc.) and, accordingly, the response of such systems will depend both on the properties of the porous matrix, which determines the growth mechanism of the nickel phase, and on the conditions for the formation of nickel or its compounds.

Impedance spectroscopy is widely used to measure the response of gas sensors. Electrochemical impedance spectroscopy has been applied to measure the H_2_S gas response of the sensor fabricated on reduced graphene oxide-incorporated nano-zinc oxide composites [32]. In [33] the complex impedance technique was established for proper adjustment of the operation frequency of the PANI-based gas sensor. Study of arrayed one-dimensional nickel nanowires using impedance spectroscopy revealed increase in resistance of the aligned nanowires at low frequencies by the adsorption of water and ethanol molecules [34]. The analysis of the dielectric permittivity spectra of macroporous silicon was carried out taking into account the structure of the material and showed the ejection of electrons from trapped states at 250 K [35]. The presence of a spectrum of localized states due to the defectiveness of the structure may suggest the existence of electrically active defects.

Thus, the aim of this work is to develop new approaches to increasing the sensitivity of adsorption gas sensors based on composites of porous silicon and nickel, as well as oxide materials in a fractal-percolation structure. In this work, layers of porous silicon functionalized with nickel, as well as zinc oxide with fractal-percolation structure were obtained. It is shown that these materials show a high response to organic solvent vapors and have the potential for use in gas sensors. A model is proposed that explains the high sensitivity values and the inductive nature of the impedance at low frequencies, taking into account the structural features and fractal-percolation properties of the obtained oxide materials.

## 2. Materials and Methods

### 2.1. Synthesis of Oxide Nanomaterials with Fractal-Percolation Structure

Nanostructured layers of ZnO were prepared via chemical coprecipitation method [36,37]. ZnO powder was synthesized from aqueous solution of Zn(CH_3_COO)_2_·2H_2_O. An aqueous solution of NH_4_OH was added to the solution under stirring until pH = 7 was reached. To remove unreacted ions, dialysis of the suspension was carried out during 24 h. Precipitated suspension was placed into the dialysis tubing which was washed from the outside with distilled water. The content of the precipitated hydroxides remained constant, since the membrane is impermeable to them, and the unreacted ions gradually diffused into the water and were removed. The precipitate was centrifuged and washed with distilled water several times, then dried for 24 h at 80 °C and annealed for 3 h at 350 °C.

### 2.2. Synthesis of Porous Silicon Layers and Their Functionalization by Nickel

Porous silicon layers were obtained by electrochemical anodic etching. Monocrystalline p-Si (111) with a resistivity of 10 Ω cm was used as a substrate. An aqueous-alcohol (isopropanol) solution of hydrogen fluoride was used as an electrolyte. Electrochemical etching of silicon wafers was carried out in a 2-chamber cell of the Unno-Imai type; the scheme of the installation is given elsewhere [38,39,40]. The choice of technological conditions for etching is due to the results of previous studies [27,31,41]. With such a crystallographic orientation of silicon (Si (111)) and relatively low anodization current densities, the anodic dissolution of silicon will occur along the directions of the fastest etching (for silicon, this is a family of directions <100>). This will make it possible to form a system of pore channels with a dendritic-fractal type of porous texture. Technological conditions for obtaining layers of porous silicon are shown in Table 1.

Porous silicon layers were functionalized with nickel by means of two techniques: impregnation and electrochemical cathode deposition. Both methods are relatively simple, cheap, do not require expensive equipment, and while they are effective for producing nanomaterials, they are also effective for the functionalization of porous materials [42,43]. An aqueous-alcohol solution of nickel chloride (1% of salt) was used for functionalization. The ratio of water and isopropanol was chosen so that the electrolyte wetted the por-Si substrates. 

The first technique of por-Si functionalization was the impregnation of porous silicon in the electrolyte described above for 7 days, then the samples were dried in air. 

Another technique for Ni intercalation into porous silicon was electrochemical cathodic deposition. Electrochemical cathodic deposition was carried out using two-electrode electrochemical cell (Pt electrodes), the deposition potential was controlled using a PI-50-Pro-3 potentiostat/galvanostat (Elins LLC, Moscow, Russia). The synthesis conditions are specified in Table 1.

### 2.3. Por-Si/Ni Morphology and Optical Properties

Surface morphology and texture of porous silicon (porous layer thickness, shape, cross-sectional diameter and pore channel configuration) was studied using Helios Nanolab D449 Dual Beam electron microscope (FEI Company, Hillsboro, OR, USA). Accelerating voltage was 10 kV, current was 0.17 nA. The image was obtained at an angle of 52 degrees.

The optical characteristics (refractive index) of Ni-containing por-Si were studied by the ellipsometry method [44]. Changes in the polarization state of electromagnetic radiation (λ = 632.8) when reflected from the sample was analyzed. The inverse task of ellipsometry method was solved (the effective optical characteristics of the layer (ε, n) were determined by the given geometric parameters of the sample (layer thickness)).

### 2.4. Impedance Spectroscopy Studies

Metal oxide powders were pressed into pellets for measurements. Electrical contacts are formed on the surface of por-Si layers and oxide pellets using contactol (3 g Art-№.530042, SILBERLEITLACK, Ferro GmbH, Frankfurt, Germany). Gas sensitive properties were studied under acetone, ethanol and isopropanol vapors exposure (1000 ppm). 

Studies of the electrical properties of sensor materials were carried out using the impedance spectroscopy method. The frequency dependences of the complex resistance module and the phase shift angle between current and voltage in a capacitive circuit in the frequency range from 100 Hz to 500 kHz were measured using an impedance meter Z500P (Elins LLC). The air is supplied to the measuring cell by a compressor through a container with silica gel to dehumidify. Then part of the dried air flow passes through the bubbler, where it is mixed with vapors of liquid (acetone, alcohol or isopropanol). The required concentration of the detected gas is set by changing the ratio of air flows (pure and with organic solvent vapors). The impedance was presented on the complex plane in the form of dependencies of the real and imaginary components of the complex resistance.

The sensor responses in the frequency range from 100 Hz to 500 kHz were calculated as S_Im_ = Im(Z)_air_/Im(Z)_gas_, where Z_air_, Z_gas_ are complex resistances in the air atmosphere and in the presence of a target gas, respectively.

## 3. Results

### 3.1. Por-Si Microstructure 

**Figure 1:**
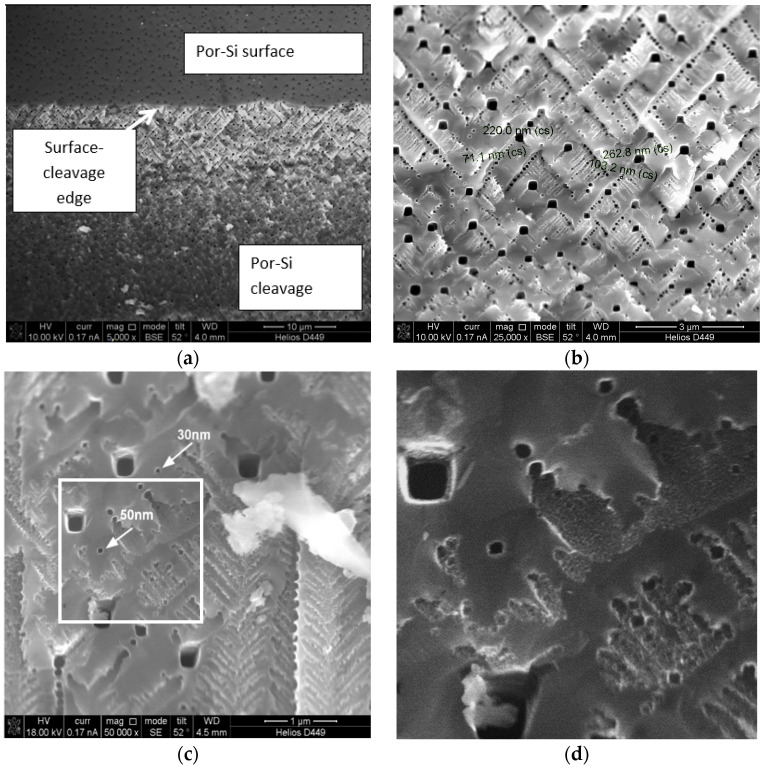
(**a**–**c**) SEM images of por-Si at different magnification (p-por-Si (111), *J*_A_ = 20 mA/cm^2^  *t* = 10 min) ((**d**) shows the fragment marked by white square marker at (**c**)).

Figure 1 shows the microstructure of a typical por-Si sample synthesized under conditions presented in Table 1. Figure 1 demonstrates the peculiar texture of the porous layer: one can observe a family of pore channels propagating at an angle of ≈45 degrees with respect to the surface; pore channels develop in the direction of the crystallographic direction that dissolves fastest (for silicon, this is <100> system of directions). At the same time, the pores demonstrate a well-defined square shape in cross-section. The pore channels show a dendritic character. According to Figure 1 at various magnifications of SEM, branching up to 4th level could be observed, while the pore sizes at different levels were: (1) 220–330 nm (macropores), (2) 60–70 nm (macropores), (3) 40–50 nm (mesopores) and (4) 20–30 nm (mesopores). The porosity is uniform in the thickness of the layer, the boundary with monocrystalline silicon is uniform, clear. The thickness of the porous layer is ≈40 microns. 

It should be noted that after the treatment of porous silicon layers in a nickel electrolyte by both techniques, according to the SEM data, no significant change in morphology was observed. At the same time, organoleptic analysis showed a change in color (from gray-black, typical for porous silicon of this series, to yellowish-greenish (after treatment in a nickel electrolyte).

### 3.2. Investigation of the Refractive Index of por-Si and por-Si/Ni

Changes in the optical properties of porous silicon as a result of its functionalization by nickel are presented in Table 2. In this case, the method is effective for indirect observation of the presence of small amounts of nickel by changing the refractive index.

It is shown that the refractive index of por-Si is close to the one of SiO_2_ because silicon is oxidized in air, and porous silicon is oxidized even more intensively as it has a large specific surface area. It is well-known that its oxide (SiO_2_, SiO_x_) is always present on the surface of por-Si in air [45].

The evaluation of the refractive index (n) of nickel-containing layers of porous silicon showed that n of such samples is more than 2.54, which is significantly higher than the values of the initial porous silicon (≈1.45–1.54). Thus, the ellipsometry method is sensitive to the presence of small amounts of the nickel-containing phase in porous silicon.

It was found that with an increase in the impregnation time of por-Si in nickel chloride solution, n increases (from n = 2.54 after 7 days to n = 3.11 after 36 days). It was suggested that nickel ions bind oxygen on the surface of porous silicon, thus the refractive approaches the one of silicon (n_Si_ = 3.4).

### 3.3. Impedance Spectroscopy of por-Si and por-Si/Ni

**Figure 2:**
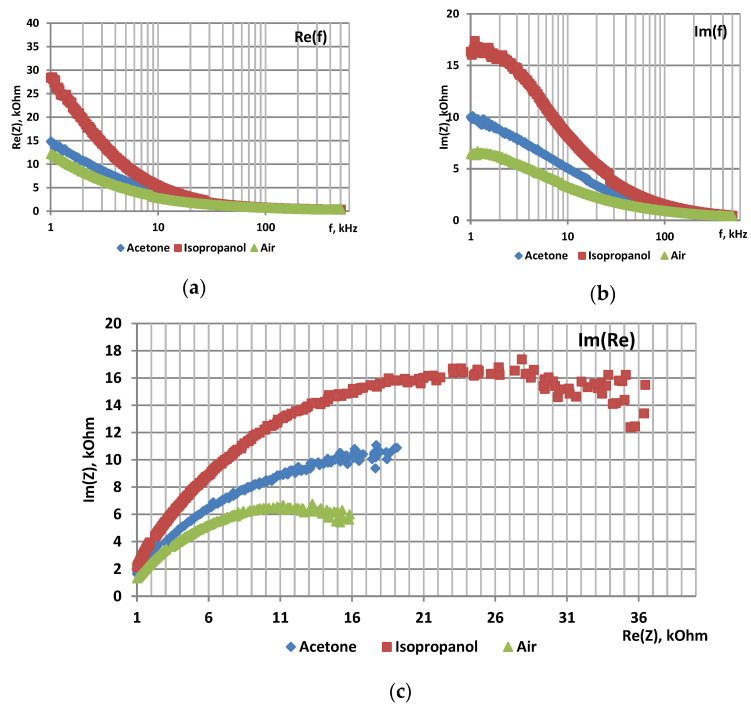
Impedance characteristics of por-Si: (**a**)—frequency dependence of Re(Z), (**b**)—frequency dependence of Im(Z), (**c**)—Nyquist plot.

**Figure 3:**
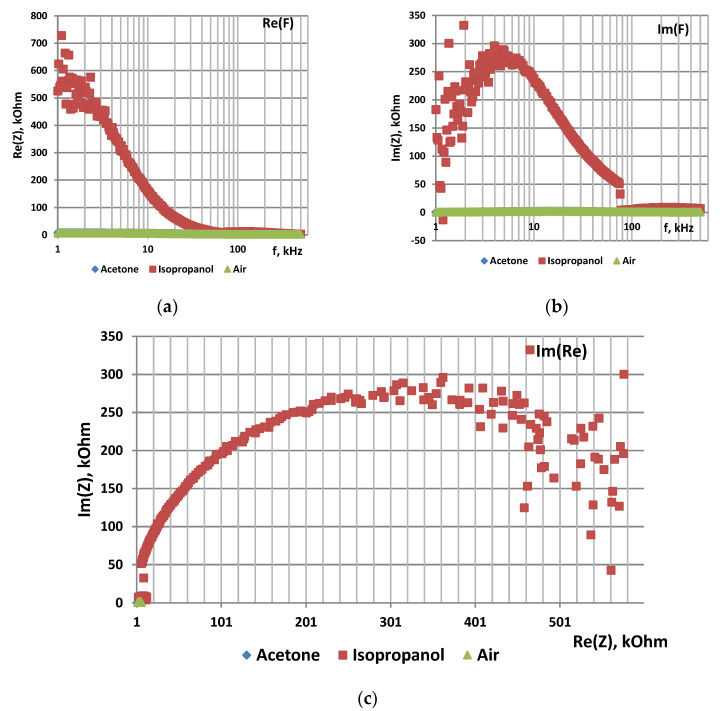
Impedance characteristics of por-Si functionalized with Ni by impregnation: (**a**)—frequency dependence of Re(Z), (**b**)—frequency dependence of Im(Z), (**c**)—Nyquist plot.

**Figure 4:**
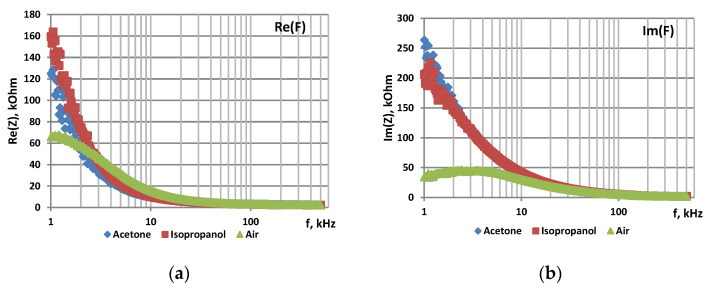

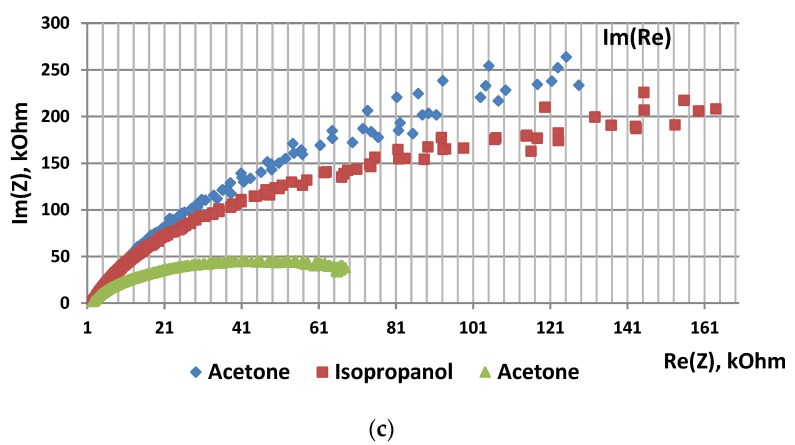
Impedance characteristics of por-Si functionalized with Ni by electrochemical deposition: (**a**)—frequency dependence of Re(Z), (**b**)—frequency dependence of Im(Z), (**c**)—Nyquist plot.

The samples were studied using impedance spectroscopy in an air atmosphere, in the presence of acetone and isopropanol vapors at 78 °C. Figure 2, Figure 3 and Figure 4 show the frequency dependences of the real and imaginary components of the impedance, as well as the Nyquist plot for por-Si and por-Si functionalized with nickel by two different methods.

The sensor responses to acetone and isopropanol vapors calculated from the imaginary part of impedance are shown in Table 3. 

It was found that both the real and imaginary parts of the impedance increase in the presence of reducing gas vapors because the initial Si is a p-type semiconductor. Besides, if NiO is formed during functionalization, it is also a p-type material semiconductor.

The sensor responses of por-Si treated with a nickel solution to various gases depend on the processing method. Por-Si/Ni, functionalized by impregnation into a nickel-containing solution, has response to acetone vapor close to the one of untreated por-Si, while its response to isopropanol vapor is significantly higher (472.73). The response of por-Si/Ni functionalized by electrochemical deposition to both gases is almost the same and several times higher than the response of por-Si. The different responces of por-Si/Ni is probably due the presence of different phases of nickel (Ni or NiO).

### 3.4. Impedance Spectroscopy of Metal Oxides with Fractal Percolation Structure

**Figure 5:**
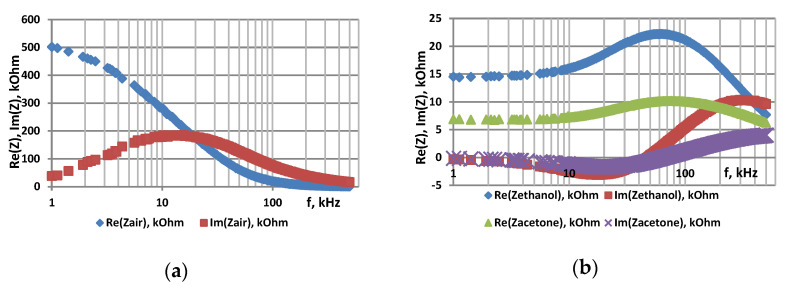
Frequency dependence of real and imaginary parts of impedance of ZnO: (**a**)—in air, (**b**)—in acetone and ethanol vapors.

Figure 5 shows changes in electric properties of zinc oxide pellet in wide frequency range in the air atmosphere (Figure 5a) and in the presence of acetone and ethanol vapors (Figure 5b) at 350 °C. 

**Figure 6:**
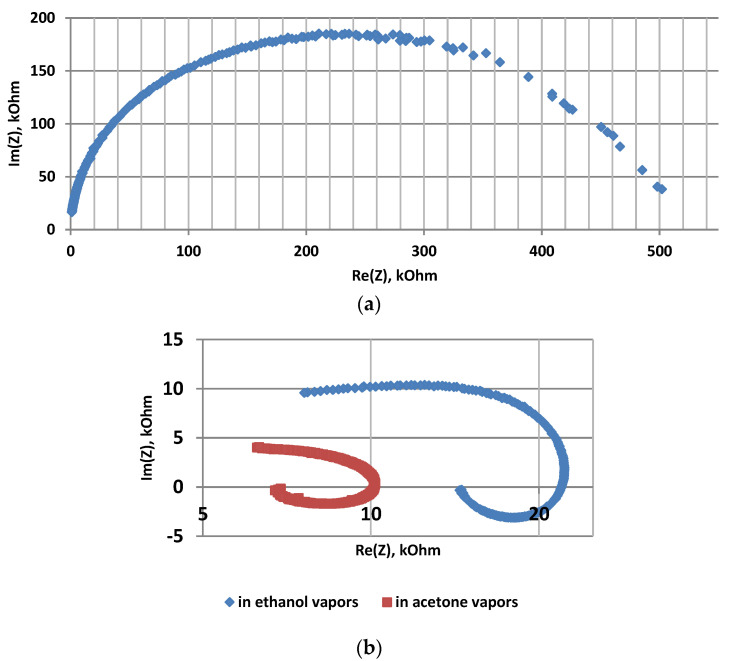
Nyquist plots of ZnO: (**a**)—in air, (**b**)—in acetone and ethanol vapors.

Figure 6 shows Nyquist plots for ZnO pellet in the air atmosphere (Figure 6a) and in the presence of acetone and ethanol vapors (Figure 6b) at 350 °C. It was revealed that the highest sensor response to ethanol vapors is 2449 at 47 Hz. The highest response to acetone vapors is 5243 at 10 Hz.

It was found that Nyquist plots in the presence of reducing gases shows inductive behavior in the low-frequency region and impedance hodographs shift in the IV quarter of the trigonometric circle. This fact is apparently related to the fractal nature of the sensor metal oxide layers, characterized by the formation of a percolation merged cluster near the percolation threshold.

### 3.5. Fractal Model of Porous Sensor Materials

The growth and assembly of grains during coprecipitation lead to the formation of pores with fractal surface [46,47]. The nature of assembly at the initial stages of synthesis is the main factor determining the emergence of fractal structures. Brownian motion of particles occurs under conditions of diffusion-limited aggregation. At co-precipitation in an aqueous medium OH-groups remain in the structure. Then they evaporate as a result of drying and annealing, leading to the formation of a porous structure.

The changes in electric properties of sensor materials are usually interpreted using the following model. Depleted layer is formed in the subsurface area with the thickness of Debye length. If the gas-sensitive layer consists of grains smaller in size than the Debye length or grains whose modulated contact area is comparable to the cross-sectional area at the grain boundary, then it has high sensor response. However, it is not possible to explain the response value to acetone within this model. 

The observed effects can be explained by the formation of a percolation merged cluster under conditions slightly exceeding the percolation threshold. The technical devices implemented at the percolation threshold are certainly of great interest [48,49,50,51]. In such structures, when interacting with oxygen, the percolation cluster breaks off. So, the gas-sensitive layer has a fractal structure before the percolation threshold and the electrical resistance of the layer can be very high. When interacting with the reducing gases, the effective conductivity will correspond to the state after the percolation threshold. Within the framework of the percolation cluster model with a structure at the percolation threshold, there are no fundamental limitations in the values of gas response.

**Figure 7:**
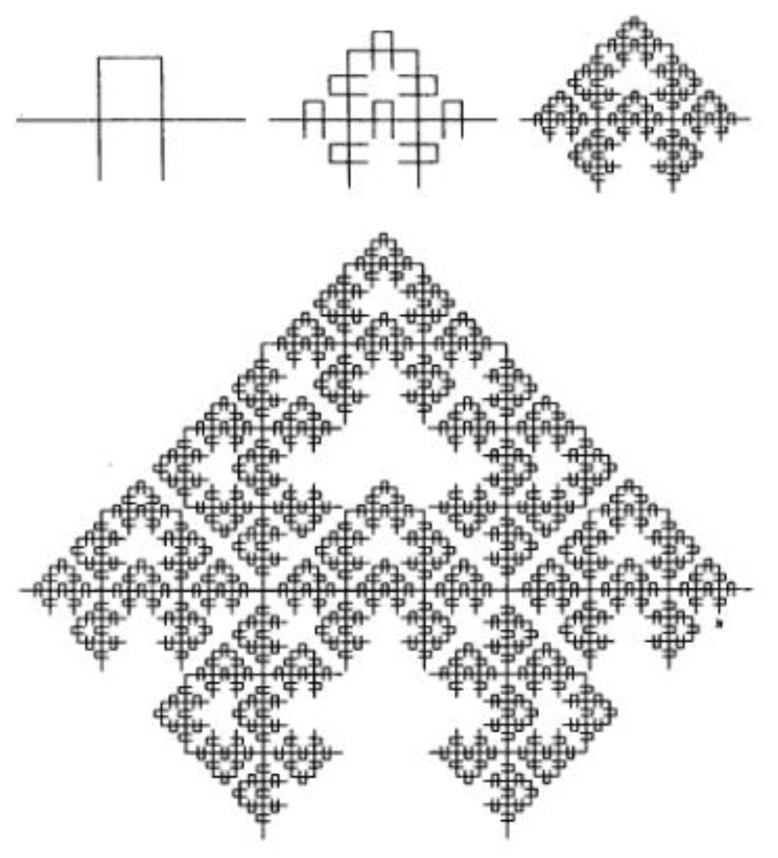
Mandelbrot-Given fractal.

According to the Mandelbrot-Given model, the fractal contains the same elements as the percolation cluster [52]. The Mandelbrot-Given model was proposed because its Hausdorff fractal dimension is close to the value corresponding to the fractal properties of an infinite cluster in two-dimensional space. The forming element for this curve divides a linear segment into parts of length r = 1/3 and connects them into a loop consisting of three parts, to which two branches are attached (Figure 7). The Mandelbrot-Given curve is interesting because it has loops and branches of all possible sizes. At each iteration, the generating element replaces each linear link with N = 8 links reduced by a third. Thus, the Mandelbrot-Given curve has a fractal dimension D = ln8/ln3 = 1.89 [53].

**Figure 8:**
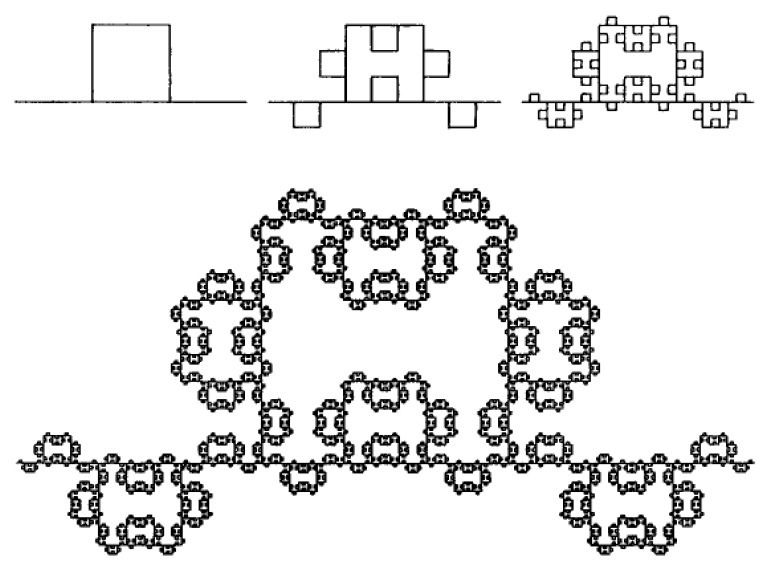
Backbone of Mandelbrot-Given curve.

The experimental results can be explained by the formation of a percolation merged cluster in a state slightly after the percolation threshold. The cluster power can be described qualitatively by the Mandelbrot-Given model. If the voltage is applied between the left and right ends of the structure shown in Figure 6, then the current flow through only through the backbone of the structure. The remaining parts are “dead ends”, which corresponds to the real structure of nanomaterials. The electric properties of a percolation cluster close to the percolation threshold are determined by the fractal properties of its backbone (Figure 8), i.e., the curve excluding branches connected to the initial linear segment with one end. The Hausdorff fractal dimension for the conducting backbone is close to the corresponding value for the Sierpinsky napkin (D = 1.62). Thus, the fractal gas-sensitive structure exists in two-dimensional space and has a topological dimension equal to one, thus the blocking of the flow paths is possible during adsorption charged oxygen ions on the surface of sensor layer.

When the material is in the air atmosphere, oxygen chemisorption occurs on its surface, which leads to blocking of some conductive fractal branches, and, as a consequence, the transition of the system to a state before the percolation threshold characterized by a high resistance. When reducing gas molecules appear, they are adsorbed on the surface of the material and react with oxygen. The reaction products are desorbed from the surface and the flow paths are unblocked. Thus, the system transfers through the percolation threshold, the current flow path length decreases and consequently resistance also significantly decreases. When the conductive branches are unblocked, random loops of all possible sizes are formed.

**Figure 9:**
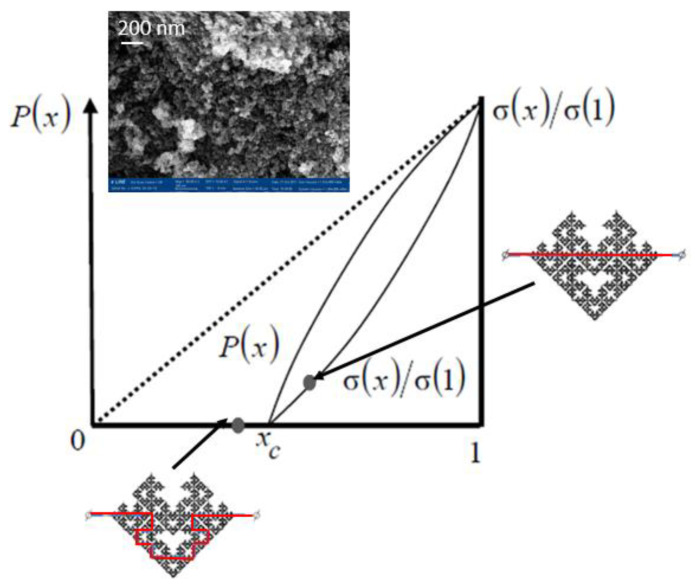
Dependences of the power and reduced conductivity of the percolation cluster.

Figure 9 illustrates two possible states of the system with different values of resistance in different atmospheric conditions. The dependence of the resistance on the fraction of conducting particles in the system can be described by the following equation:

R ~ 1/(x − x_c_)^t^,

where t is the critical index of electrical conductivity (t_2_ = 1.3 for the space dimension d = 2 and t_3_ = 1.6–1.7 for d = 3), x_c_ is the percolation threshold. It should be noted that in real nanomaterials in the state close to the percolation threshold x_c_ value is variable, so gas-sensitive properties are instable and can be different for similar samples.

As can be seen from the model scheme of the Mandelbrot-Given percolation cluster, system includes loops of various diameters. At high frequencies, the loops almost do not participate in the charge carrier transport, since they have inductive character. At low frequencies, on the contrary, loop paths become more preferred compared to capacitive ones, which in real structure are dielectric layers between conductive semiconductor grains. Thus, the inductive nature of impedance spectroscopy at low frequencies follows from the above scheme. After long annealing or at higher concentration of precursor solution the fractal character disappears.

Of course, this model only qualitatively describes properties of gas sensor layers. In addition, in the model, the appearance of Debye depleted region still remains. Thus, it develops well-known models, expanding the range of effects explained. Blocking the current flow paths involves the exclusion of branches with a cross-section less than the effective Debye length. This model also confirms that in adsorption semiconductor sensors with a fractal structure, the gas response can significantly exceed the highest value according to model with classical modulation of the conductivity of sub-surface layers.

## 4. Conclusions

The fractal-percolation structures based on nanostructured layers of zinc oxide and layers of porous silicon functionalized with nickel were obtained. Meso-/macroporous silicon with fractal-dendritic type of pore channels (with at least four levels of hierarchical branching pores) were obtained by electrochemical anode etching of p-SI (111). The treatment of porous silicon layers with an aqueous isopropanol solution of nickel chloride was carried out by two techniques: the impregnation method and electrochemical cathodic deposition. It is shown by SEM data that the treatment of porous silicon in a nickel solution (by either technique) does not lead to noticeable changes in the morphology of the porous layer, nevertheless, the refractive index and impedance characteristics of such samples change noticeably both in air and in the presence of organic volatile substances (acetone, isopropanol). 

Nickel deposition in porous silicon increases the sensitivity of the samples to isopropyl alcohol vapors. Moreover, the sensitivity (the contribution to the impedance due to the interaction with nickel) is greater, when the processing time for samples obtained by both methods is longer.

The highest response of ZnO to acetone vapors was 5243 at 10 Hz, while to ethanol vapors it is 2449 at 47 Hz. The obtained gas sensing response values are explained within the framework of a model that takes into account the fractal nature of nanostructured powders and the transition through the percolation threshold in an atmosphere containing a reducing gas.

## Figures and Tables

**Table 1:** Technological conditions for obtaining por-Si and its functionalization by Ni.

No	Por-Si Synthesis Conditions		Ni Functionalization Conditions			
	J_A_, mA/sm^2^	t, min	Method	Electrolyte/Solution	U, V	t, min
I	20	10	Electro-chemical deposition	1:10:10 NiCl_2_:H_2_O: C_3_H_7_OH	10	30
II					15	5
III			Impregnation		-	Impregnation time

**Table 2:** Refractive index of por-Si and por-Si/Ni.

Sample	Ni Functionalization Method	Impregnation Time, Days	Refractive Index
Por-Si	Impregnation	-	1.414
		-	1.586
Por-Si/Ni		7	2.540
		21	2.723
		36	3.107

**Table 3:** Maximum values of por-Si and por-Si/Ni sensor responses.

Sample	Acetone		Isopropanol	
	S	f, Hz	S	f, Hz
Por-Si	1.58	1052	2.68	1103
Por-Si/Ni (impregnation)	1.28	1839	472.73	1359
Por-Si/Ni (electrochemical cathode deposition)	7.64	1005	6.24	1155

## Data Availability

The data presented in this study are available on request from the corresponding author.
